# Chronic Exposure to Rhodobacter Sphaeroides Extract Lycogen™ Prevents UVA-Induced Malondialdehyde Accumulation and Procollagen I Down-Regulation in Human Dermal Fibroblasts

**DOI:** 10.3390/ijms15021686

**Published:** 2014-01-23

**Authors:** Tsai-Hsiu Yang, Ying-Hsiu Lai, Tsuey-Pin Lin, Wen-Sheng Liu, Li-Chun Kuan, Chia-Chyuan Liu

**Affiliations:** 1 Department of Health and Nutrition, Chia-Nan University of Pharmacy and Science, Tainan 71710, Taiwan; E-Mails: connie@mail.chna.edu.tw (T.-H.Y.); tplin007@mail.chna.edu.tw (T.-P.L.); lichunkuan@hotmail.com (L.-C.K.); 2 Department of Medical Research and Education, Taipei Veterans General Hospital, Taipei 11217, Taiwan; E-Mail: d49405004@gmail.com; 3 Asia-Pacific Biotech Developing, Inc., Kaohsiung 80681, Taiwan; E-Mail: wensheng5394@gmail.com; 4 Department of Marine Biotechnology and Resources, National Sun Yat-Sen University, Kaohsiung 80424, Taiwan; 5 Department and Institute of Cosmetic Science, Chia-Nan University of Pharmacy and Science, Tainan 71710, Taiwan

**Keywords:** Lycogen™, skin aging, UVA, procollagen I, matrix metalloproteinase

## Abstract

UVA contributes to the pathogenesis of skin aging by downregulation of procollagen I content and induction of matrix metalloproteinase (MMP)-associated responses. Application of antioxidants such as lycopene has been demonstrated as a convenient way to achieve protection against skin aging. Lycogen™, derived from the extracts of *Rhodobacter sphaeroides*, exerts several biological effects similar to that of lycopene whereas most of its anti-aging efficacy remains uncertain. In this study, we attempted to examine whether Lycogen™ could suppress malondialdehyde (MDA) accumulation and restore downregulated procollagen I expression induced by UVA exposure. In human dermal fibroblasts Hs68 cells, UVA repressed cell viability and decreased procollagen I protein content accompanied with the induction of MMP-1 and MDA accumulation. Remarkably, incubation with 50 μM Lycogen™ for 24 h ameliorated UVA-induced cell death and restored UVA-induced downregulation of procollagen in a dose-related manner. Lycogen™ treatment also prevented the UVA-induced MMP-1 upregulation and intracellular MDA generation in Hs68 cells. Activation of NFκB levels, one of the downstream events induced by UVA irradiation and MMP-1 induction, were also prevented by Lycogen™ administration. Taken together, our findings demonstrate that Lycogen™ may be an alternative agent that prevents UVA-induced skin aging and could be used in cosmetic and pharmaceutical applications.

## 1. Introduction

In addition to serving as the largest organ of the body, the skin is one of the first defense mechanisms of the immune system. Skin immunity allows skin itself to resist infections from pathogens and noxious stimuli. Skin ageing is a complex process and can be influenced by both intrinsic and extrinsic factors [1]. Among all extrinsic factors, UV-light exposure is the most common one [2]. Based upon its wavelength, UV light penetrates into the skin and affects different cells. UVB (290–320 nm), predominantly absorbed by the epidermis, may trigger sunburns. UVA (320−400 nm) penetrates deeper to the dermis where collagen is located and may subsequently decrease collagen production [1]. Overall, UVB mainly interacts with keratinocytes and other epidermal cells, but UVA interacts with both epidermal and dermal cells such as human dermal fibroblasts. Actually, UVA has been demonstrated to decrease type I collagen [3,4], the most abundant collagen, forming more than 90% of the dry weight of dermis and providing structural support [1], and inhibits the biosynthesis of pro-collagen synthesis in human dermal fibroblasts [3,4]. It was widely accepted that UVA triggers photoaging via two major pathways, *i.e.*, induction of matrix metalloproteinases (MMPs) and mutations in mitochondrial DNA [5]. The resultant generation of reactive oxygen species (ROS) has been shown to mediate effects including transcription factor activation, lipid peroxidation [6], and DNA double-strand breaks [7]. Collectively, prevention of UV light-induced skin ageing, especially that induced by UVA, is urgently needed for anti-ageing therapy.

Many phytonutrients have been described as promising photoprotectants in cell culture, animal and clinical studies [8]. In regard to skin health, these phytonutrients include vitamin E, certain flavonoids, and the carotenoids, β-carotene, lycopene and lutein [8]. So far, several natural products (e.g., plant/herbal products) and strategies against skin aging have been developed. The possible mechanisms of these natural products/strategies may involve scavenging free radicals, protecting the skin matrix via the inhibition of enzymatic degradation, or promoting the synthesis of collagen in the skin [9]. Lycopene, a member of the carotenoid family, is the red-colored pigment predominantly found in red colored fruits and vegetables such as tomato, papaya, watermelon, *etc.* It has been shown that cutaneous concentration of lycopene correlates with the roughness of the skin [10]. Actually, some evidence further demonstrated that topical application of lycopene is a convenient way to restore the UV-depleted antioxidants from the skin and achieve skin protection against premature aging and cancer [11]. In addition to these protective effects on skin, it has also been shown that lycopene exhibits antioxidant and anti-inflammatory actions in macrophages [12], inhibits angiogenesis *in vitro* and *in vivo* [13] and decreases cancer risk [14], and may provide protection against cardiovascular diseases [15]. Although several lines of evidence demonstrated the therapeutic potential of lycopene, several limitations hindered the development and clinical utility of lycopene. For example, lycopene is hydrophobic but highly soluble in organic solvents. Extraction of lycopene using organic solvents is usually toxic, expensive, and hazardous. Alternatively, using supercritical CO_2_ as a solvent to extract lycopene from waste tomato skin carries less toxicity and other hazards. Nevertheless, the dissolution of lycopene in supercritical CO_2_ is still compelling, and its measurement is difficult due to its instability [16]. Therefore, it is urgent to develop reliable methods to extract lycopene or identify novel bioactive compounds that exhibit functions similar to that of lycopene.

Microorganisms such as fungi and bacteria may produce metabolites that have been used in cosmetic and pharmaceutical applications. Some of these bacteria-derived metabolites/compounds have been shown to possess prominent therapeutic potentials that may have clinical utility. *Rhodobacter sphaeroides* is a group of bacteria that can generate energy through photosynthesis. An extract of *Rhodobacter sphaeroides*, named Lycogen™ is a dark-red compound. The patent strain (WL-APD911) of Lycogen™ was developed by Asia-Pacific Biotech Developing Inc. (Kaohsiung, Taiwan). Lycogen™ has attracted significant attention due to its dramatic biotechnological availability [17,18]. For example, Lycogen™ can serve as an anti-inflammatory agent that ameliorates dextran sodium sulfate-induced colitis in mice [17]. In addition, it also inhibits melanogenesis through the MEK/ERK signaling pathway [18]. UVA leads to various unfavorable outcomes of skin biology and is widely known to contribute to the development of skin aging. Although Lycogen™ possesses several biological actions similar to that exerted by lycopene, the known phytoprotectant in skin health, it still remains an open question whether Lycogen™ also protects skin from noxious insult caused by UVA. In this study, we attempted to investigate whether administration of the extract of *Rhodobacter sphaeroide* Lycogen™ could prevent the decreased cell viability, downregulation of procollagen I, the activation of MMP-associated pathways, and accumulation of the products of lipid peroxidation, which are largely induced or recruited by UVA exposure.

## 2. Results and Discussion

### 2.1. Toxicity Testing of Lycogen™ in Human Fibroblast Lines

**Figure 1.**
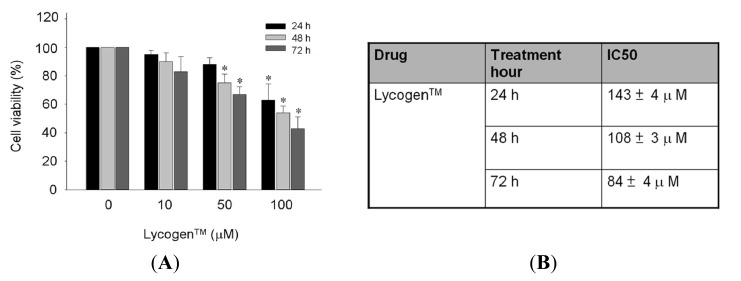
Toxicity testing of Lycogen™ for cell viability (**A**) and IC50 (**B**) in human fibroblast Hs68 cells. Hs68 cells were treated with various doses of Lycogen™ (dissolved in THF) for the indicated time. Cell viability was determined by MTT assay and was expressed as percent viable cells in the total number of cells counted. Data shown here are the mean ± SD of at least three independent experiments. ^*^ *p* < 0.05 *vs.* 0 μM (THF alone) at the same indicated time.

Prior to examining the potential utility of Lycogen™ in the treatment of skin abnormalities induced by UV irradiation, we first evaluated whether Lycogen™ affected skin dermis viability. To determine the toxic effects of Lycogen™ on human dermal fibroblasts, Lycogen™ was sequentially diluted with Tetrahydrofuran (THF) and then added to the human dermal fibroblast lines Hs68 cells and cultured for 24, 48 or 72 h. A MTT assay was used to analyze the viability of the Hs68 cell lines, in response to Lycogen™ treatment. No treatment effect was observed in cells treated with 10 μM Lycogen™ for any given time. After 24-h treatment of various doses of Lycogen™, an inhibitory effect on cell viability was observed only at the highest dose 10 μM (Figure 1A). A mild decrease in cell viability was initially observed under treatment of 50 μM Lycogen™ for 48 h, and this viability further declined by treatment for 72 h (Figure 1A). Exposure of Hs68 cells to 100 μM Lycogen™ robustly decreased cell viability in a time-dependent manner (Figure 1A). The IC_50_ of Lycogen™ treatment for 24, 48 and 72 h were 143 ± 4, 108 ± 3 and 84 ± 4 μM, respectively (Figure 1B). Based on these results, incubation with 50 μM Lycogen™ for 24 h was selected as the optimal treatment dose of Lycogen™ in Hs68 cells.

### 2.2. UVA Irradiation-Induced Cell Death Accompanied by Downregulation of Type 1 Procollagen in Human Fibroblast Lines

**Figure 2.**
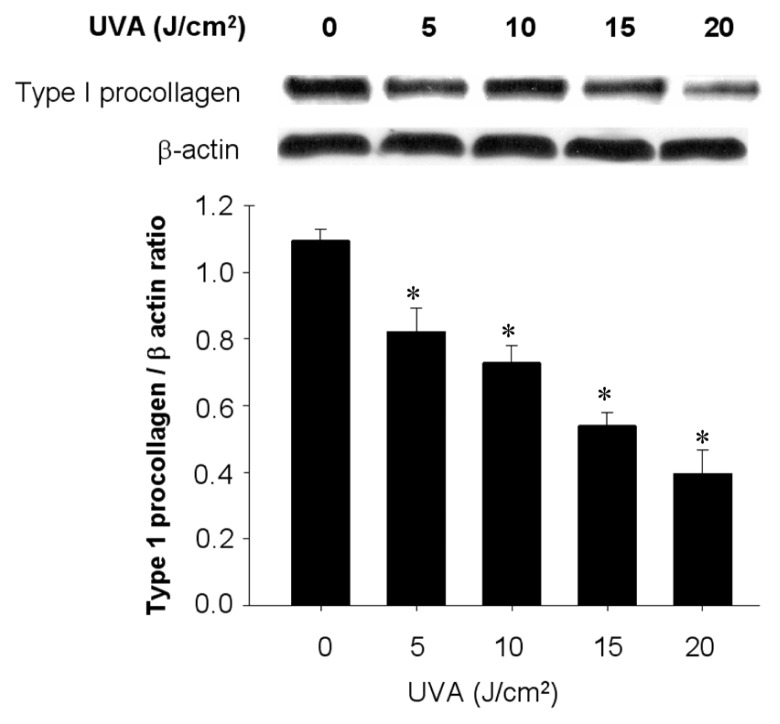
Dose-dependent inhibition of UVA irradiation on type I procollagen expression in human skin fibroblast cells. The expression of type I procollagen in human skin fibroblast cells (Hs68 cells) followed by treatment of various doses of UVA. The cells were then assigned for Western blotting. Data shown here are the mean ± SD of at least three independent experiments. ^*^ *p* < 0.05 *vs.* UVA 0 J/cm^2^.

Before elucidating the protective potential of Lycogen™ in human dermal fibroblasts Hs68 cells, we first evaluated the treatment effect of UVA irradiation on dermal fibroblast viability and the expression of type 1 procollagen, the precursor of type 1 collagen that predominantly forms the structural support of dermis [1]. Notably, exposure to UVA induced cell death in Hs68 cells in a dose-dependent manner (*LD*_50_: 55 J/cm^2^), in which a 20% decrease of cell viability was induced by UVA at the maximal dose 20 J/cm^2^ UVA (data not shown). Importantly, exposure to UVA also led to a dose-dependent decrease in the protein content of type I procollagen that was reduced ~60% by the maximal dose of UVA (Figure 2). In the subsequent experiments, we chose the fixed dose 20 J/cm^2^ of UVA as the condition of UVA irradiation in Hs68 cells.

### 2.3. Lycogen™ Ameliorated Cell Death Induced by UVA Irradiation in Human Fibroblast Lines

**Figure 3.**
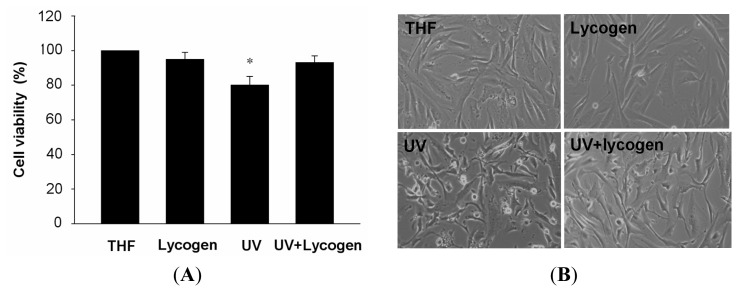
(**A**) Cell viability and (**B**) microscopic examination showing the cytoprotective effect on UVA-treated human skin fibroblast cells (Hs68 cells). Briefly, the cells were pre-incubated with 50 μM Lycogen™ for 24 h, followed by washing with PBS and further UVA irradiation with 20 J/cm^2^ UVA, than harvested after 24 h. This experiment was repeated at least three times. ^*^ *p* < 0.05 *vs.* THF.

We next examined the treatment efficacy of Lycogen™, the extracts of *Rhodobacter sphaeroides*, on Hs68 cells, with or without subsequent exposure to UVA. Chronic incubation of Hs68 cells to THF alone or 50 μM Lycogen™ for 24 h did not affect cell viability in normal Hs68 cells (Figure 3A). Exposure of Hs68 cells to UVA consistently led to an ~20% decrease in cell viability accompanied by obvious morphological changes (Figure 3A,B). Remarkably, preincubation with 50 μM Lycogen™ for 24 h completely prevented this UVA-induced cell death and ameliorated the UVA-induced morphological changes (Figure 3A,B). These results indicated that chronic incubation with Lycogen™ induced a prominent cytoprotective effect against UVA irradiation in human dermal fibroblasts.

### 2.4. Chronic Exposure to Lycogen™ Prevented UVA-Induced Downregulation of Type I Procollagen in Human Dermal Fibroblasts

**Figure 4.**
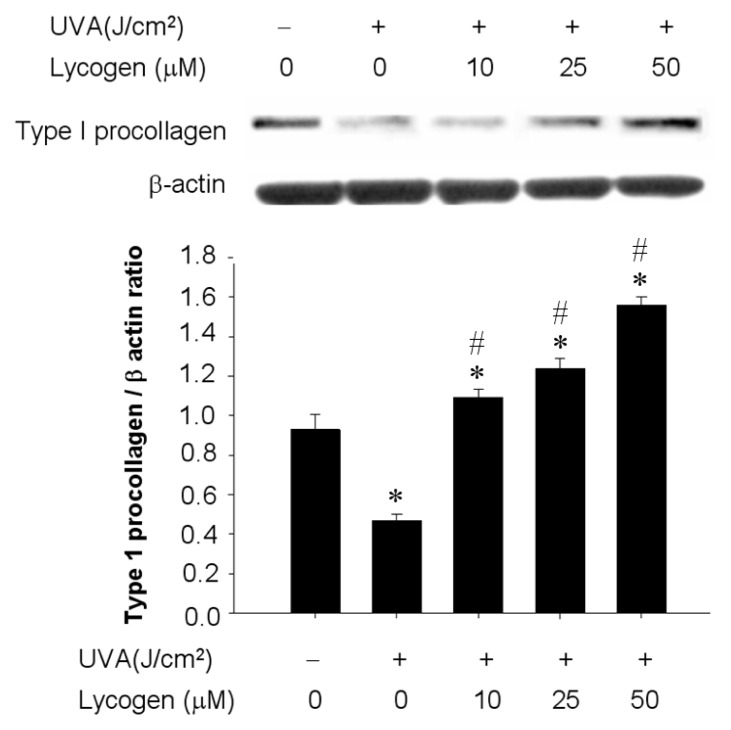
Prevention of UVA-induced downregulation of type I procollagen by Lycogen™. Hs68 cells were pre-incubated with 0, 10, 25, or 50 μM Lycogen™ for 24 h, followed by washing with PBS and further UVA irradiation with 20 J/cm^2^ UVA (+) or without UVA irradiation (−). Data shown here are the mean ± SD of at least three independent experiments. ^*^ *p* < 0.05 *vs.* 0 μM (THF alone). # *p* < 0.05 *vs.* UVA alone.

UVA has been shown to suppress type I collagen and strongly linked to the pathogenesis of skin aging. Considering the cytoprotective potential of Lycogen™, we examined whether administration of Lycogen™ could ameliorate the UVA-downregulated expression of type I procollagen, the precursor of type 1 collagen. Hs68 cells were pre-incubated with different doses of Lycogene™ for 24 h, followed by UVA irradiation with 20 J/cm^2^ UVA. Exposure of Hs68 to UVA led to a 60% decrease in type I procollagen protein content (Figure 4). Preincubation with 10, 25, or 50 μM Lycogen™ for 24 h prevented this UVA-induced downregulation of type I procollagen in a dose-dependent manner in Hs68 cells (Figure 4). These results demonstrated that chronic incubation to Lycogen™ prevented UVA-induced downregulation of type I procollagen in human dermal fibroblasts.

### 2.5. Chronic Exposure to Lycogen™ Prevented the UVA-Induced Upregulation of MMP-1 in Human Dermal Fibroblasts

**Figure 5.**
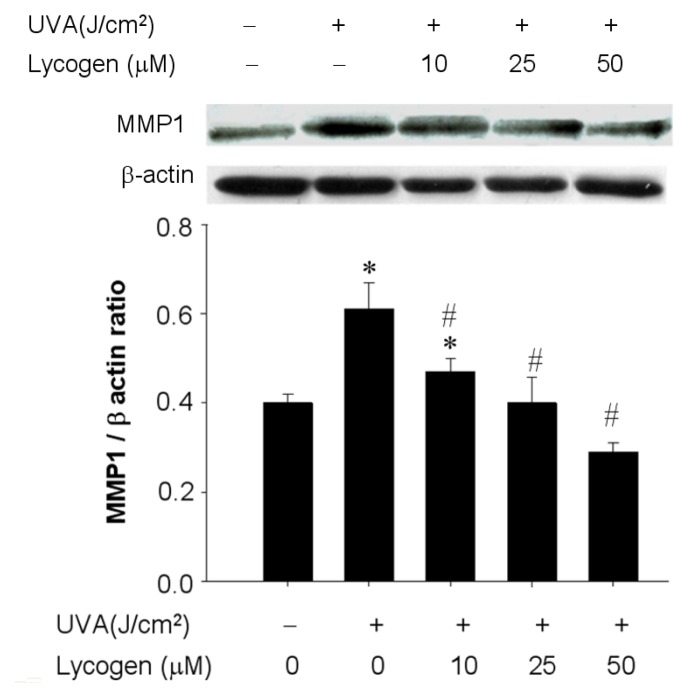
Lycogen™ prevented UVA-induced upregulation of MMP-1 in Hs68 cells. Hs68 cells were pre-incubated with 0, 10, 25, or 50 μM Lycogen™ for 24 h, followed by washing with PBS and further UVA irradiation with 20 J/cm^2^ UVA (+) or without UVA irradiation (−). The cells were then assigned for Western blotting. Data shown here are the mean ± SD of at least three independent experiments. ^*^ *p* < 0.05 *vs.* 0 μM (THF alone). # *p* < 0.05 *vs.* UVA alone.

UVA causes photoaging via the induction of MMPs that lead to the propagation of various signaling pathways and several downstream effects [6,7]. Accordingly, we examined whether Lycogen™ administration could prevent this MMP-1 upregulation induced by UVA irradiation. Twenty-minutes of UVA irradiation moderately increased MMP-1 protein expression by 1.5-fold in Hs68 cells (Figure 5). Preincubation with 25 or 50 μM Lycogen™ for 24 h prior to UVA irradiation effectively inhibited this UVA-stimulated MMP-1 upregulation in a dose-dependent manner in Hs68 cells (Figure 5). These results demonstrated that chronic Lycogen™ treatment prevented UVA-induced upregulation of MMP-I in human dermal fibroblasts.

### 2.6. Chronic Exposure to Lycogen™ Suppressed the UVA-Induced Malondialdehyde Accumulation in Human Dermal Fibroblasts

**Figure 6.**
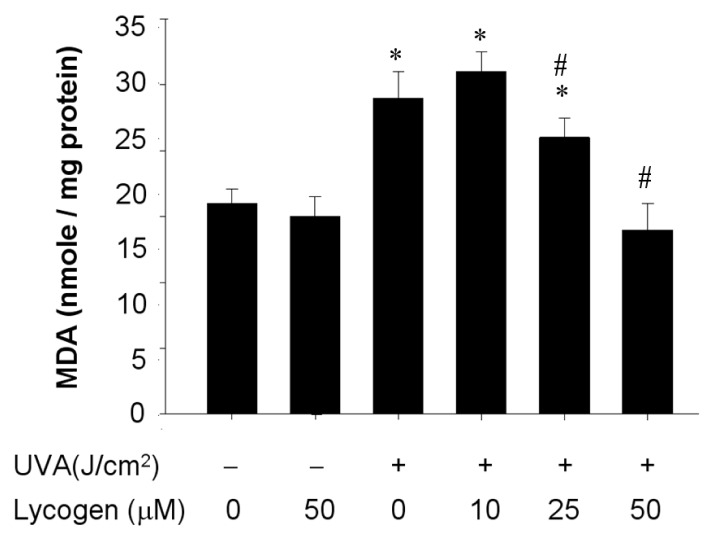
Downregulation of UVA-induced MDA accumulation by Lycogen™ in Hs68 cells. Hs68 cells were pre-incubated with 0, 10, 25, or 50 μM Lycogen™ for 24 h, followed by washing with PBS and further UVA irradiation with 20 J/cm^2^ UVA (+) or without UVA irradiation (−). The cells were then assigned for MDA determination. Data shown here are the mean ± SD of at least three independent experiments. ^*^ *p* < 0.05 *vs.* 0 μM (THF alone). # *p* < 0.05 *vs.* UVA alone.

UVA-induced MMP-1 upregulation may lead to a variety of responses, such as lipid peroxidation [6,7], and malondialdehyde (MDA) is a product of lipid peroxidation as well as one of the indicators of oxidative damage [19]. After validation of Lycogen™ effect on MMP-1 expression, we next examined whether Lycogen™ could suppress UVA-induced MDA accumulation in human dermal fibroblasts. Lycogen™ alone had no effect on MDA accumulation (Figure 6). UVA irradiation for 20 minutes resulted in acute accumulation of MDA. Hs68 cells pre-incubated with different doses of Lycogen™ for 24 h prior to UVA irradiation decreased UVA-induced MDA production in a dose-dependent manner. Lycogen™ at 50 μM led to the maximal suppression on UVA-induced MDA production (Figure 5). These results indicate that chronic Lycogen™ administration inhibited UVA-induced MDA accumulation in human dermal fibroblasts.

### 2.7. Chronic Exposure to Lycogen™ Inhibited the UVA-Induced Upregulation of NFκB Levels in Human Dermal Fibroblasts

**Figure 7.**
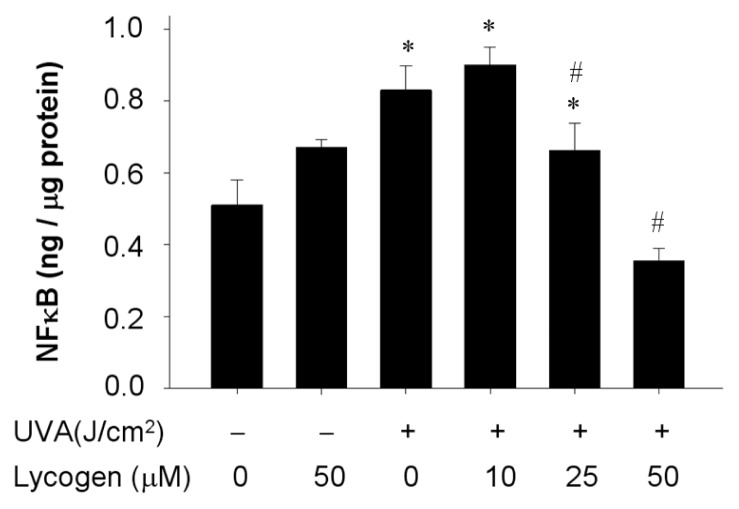
Downregulation of UVA-induced NFκB levels by Lycogen™ in Hs68 cells. Hs68 cells were pre-incubated with 0, 10, 25, or 50 μM Lycogen™ for 24 h, followed by washing with PBS and further UVA irradiation with 20 J/cm^2^ UVA (+) or without UVA irradiation (−). The cells were then assigned for NFκB levels determination. Data shown here are the mean ± SD of at least three independent experiments. ^*^ *p* < 0.05 *vs.* 0 μM (THF alone). # *p* < 0.05 *vs.* UVA alone.

Activation of transcription factors is one of downstream events induced by UVA irradiation and MMP-1 upregulation [6,7]. Accordingly, we attempted to evaluate whether Lycogen™ also affects NFκB levels in response to UVA irradiation in human dermal fibroblasts. Administration of Lycogen™ alone did not affect NFκB levels (Figure 7). UVA irradiation robustly elevated NFκB levels. Remarkably, as cells were pre-incubated with various concentrations of Lycogen™ for 24 h, the subsequent UVA-induced elevation of NFκB levels were significantly decreased in a dose-dependent manner (Figure 7). These results demonstrated that chronic Lycogen™ administration blunted the UVA-induced elevation of NFκB levels in human dermal fibroblasts.

### 2.8. Discussion

Carotenoids are a widely distributed group of naturally occurring pigments, and there are more than six hundred carotenoids that have been isolated from natural sources. The natural sources of carotenoids are usually red, orange, or yellow in color. Since carotenoids cannot by synthesized by humans, the intake of carotenoids in human largely depends on dietary intake for these micronutrients [20]. It has been demonstrated that fruits and vegetables constitute the major sources of carotenoid in the human diet [21–23]. Approximately 90% of the carotenoids in the diet and human body is represented by β-carotein, α-carotein, lycopene, lutein and cryptoxanthin [24]. Lycopene, a natural phytochemical pigment synthesized by plants and microorganisms but not by animals, has been demonstrated as a potent antioxidant and the most significant scavenger in the carotenoid family [20]. Recent data have demonstrated that lycopene can induce anti-oxidant and anti-inflammatory effect in macrophages [12] and suppress angiogenesis *in vivo* and *in vitro* [13]. Although the photoprotective efficacy of lycopene is not comparable to the use of a sunscreen, there is evidence that lycopene protects the skin against sunburn by increasing the basal defense against UV light-induced damage [25]. Unexpectedly, the clinical potential of lycopene faced several obvious limitations, including its hydrophobic property, extraction using toxic and hazardous solvent, and dissolution in supercritical CO_2_ [16]. Accordingly, the identification of novel compounds which possess lycopene-like actions was urgent and has gradually drawn great attention.

Several lines of evidence have demonstrated the potential availability of bacteria-derived metabolites in cosmetic and pharmaceutical applications [26]. For example, actinobacteria isolated from the marine environment have received considerable attention due to the structural diversity and their metabolites show a range of biological activities such as anti-bacterial, anti-tumor, and cytotoxic effects, *etc*. [26]. As for carotenoids, a group of colored terpenoids with antioxidant properties, considerable efforts have been made in the selection of micro-organisms that can provide a cost-effective source of these compounds [27]. In some bacteria, such as *Escherichia coli* and *Saccharomyces cerevisiae*, which are capable of synthesizing carotenoids naturally, *de novo* carotenoid biosynthesis has been performed by the introduction of carotenogenic genes [28,29]. The extract of *Rhodobacter sphaeroides* named Lycogen™ is a dark-red compound that has been shown to exert anti-inflammatory actions in an experimental mouse model of colitis [17] and anti-melanogenesis effect in α-MSH-treated B16F10 melanoma cells and zebrafish [18]. In this study, our findings demonstrated that the *Rhodobacter Sphaeroides* extract Lycogen™ could prevent UVA-induced malondialdehyde accumulation and procollagen I downregulation in human dermal fibroblasts, suggesting the potential utility of the *Rhodobacter Sphaeroides* extract against UVA-induced photoaging.

Lycogen™, the extract of *Rhodobacter Sphaeroides*, is a novel compound identified recently, and this agent has been demonstrated to exert several biological actions [17,18]. In addition to its anti-inflammatory effect in a mouse colitis model [17], a recent study also revealed its anti-melanogenesis effect, highlighting its potential in cosmetic and pharmaceutical applications. Considering the contribution of UVA to photoaging, remarkable efforts have been made to elucidate the mechanisms through which various agents protect the skin from UVA-induced photoaging. Prevention of MMP-1 induction, which may elicit several downstream events unfavorable to skin biology, has been demonstrated to be the major target in the treatment of UVA-induced skin damage [30–33]. For example, hydrogen-rich electrolyzed warm water (HW) has been demonstrated to repress UVA-induced skin damage by ROS-scavenging, and stimulate the synthesis of type-I collagen in dermis [32]. Fermented Citrus Unshiu peel extract also can protect human dermal fibroblasts against UVA-induced photoaging by suppressing MMP-1 gene expression and the proportion of senescence-associated β-galactosidase (SA-β-gal) [30]. Compound K, one of the major metabolites of ginsenosides, has also been shown to prevent UVA-mediated MMP-1 induction and photoaging [31]. Generation of oxidative stress substance is a downstream event of UVA-mediated MMP-1 induction [32], which has been shown to mediate several effects such as transcription factor activation, lipid peroxidation [6], and DNA single-strand breaks [7]. In addition, UVA irradiation was shown to induce changes in the p53-dependent NFκB complex that leads to growth arrest and apoptosis through the repression of cyclin D1 [34]. In this study, our findings have demonstrated the novel actions induced by the *Rhodobacter Sphaeroides* extract Lycogen™, which may protect skin dermal fibroblasts by increasing procollagen 1, and suppress the induction of MMP-1. Various MMP-1 downstream events including intracellular MDA accumulation and elevated NFκB levels were efficiently prevented by Lycogen™ pretreatment. In future investigations, it will be necessary to evaluate the toxicology test and therapeutic efficacy of Lycogen™ in animal studies *in vivo*. Studies such as these may help determine the feasibility and potential of this agent for clinical use.

## 3. Materials and methods

### 3.1. Reagents

Tetrahydrofuran (THF), Trichloroacetic acid (TCA), thiobarbituric acid (TBA), butylated hydroxytoluene (BHT), MTT [3-(4,5-dimethylthiazol-2-yl)-2,5-diphenol tetrazloium bromide], deoxycytidine (dc) 5-methyldeoxycytidine (5-mdc) were from Sigma Chemical Co. (St. Louis, MO, USA). Dulbecco’s Modified Eagle Medium (DMEM), fetal bovine serum (FBS), trypsin, penicillin, streptomycin, sodium pyruvate, and non-essential amino acids (NEAA) were from GIBCO/BRL (Rockville, MD, USA). All chemicals used were of reagent or higher grade.

### 3.2. Lycogen™, Cell Culture and UVA Irradiation

*R. sphaeroides* (WL-APD911) was isolated from mutants using chemical mutagenesis (Bioresource Collection and Research (BCRC), Hsinchu, Taiwan). The *R. sphaeroides* was cultured in broth. After harvesting, the bacterial broth was centrifuged and washed with ethanol. The bacterial residue is extracted with acetone and then centrifuged by 7500 rpm for 5 min. The supernatant is filtered through filter paper and a 0.2 μm filter into a round-bottomed flask. The color of the final supernatant is dark red. Acetone is removed completely in an oven at 55 ºC. The extract of *R. sphaeroides* was named Lycogen™. Lycogen™ is available from Asia-Pacific Biotech Developing, Inc. (Kaohsiung, Taiwan). Hs68 cells (human fibroblast cells) used in this study were obtained from Bioresource Collection and Research Center, BCRC (BCRC, Hsinchu, Taiwan). The cells were grown in DMEM containing 10% (*v*/*v*) FBS, 0.12% NaHCO_3_, penicillin (100 U/mL), streptomycin (100 U/mL), and 5% CO_2_ in an incubator at 37 ºC. For each cell line, a T-75 flask was seeded with 1 × 10^6^ cells, and cells were incubated at 37 ºC. The cells were harvested at ca. 90% confluence (10^6^ cells/flask), and the survival rates were always higher than 95% by Trypan-blue assay. Cell were then incubated with Hcy or SAH at 37 ºC for 24~72 h. Lycogen™ was dissolved in THF, the final concentration of THF was 0.2%. All assays were performed in triplicate by using three flasks for each cell line. Then cells in 10-cm^2^ dishes were washed and then covered with 10 mL of Hanks balanced salt solution (1.3 mM CaCl_2_, 5.4 mM KCl, 0.4 mM KH_2_PO_4_, 0.5 mM MgCl_2_ 6H_2_O, 0.4 mM MgSO_4_ 7H_2_O, 136.7 mM NaCl, 4.2 mM NaHCO_3_ and 0.3 mM NaH_2_PO_4_ H_2_O).

Irradiation was carried out in a UVA irradiation chamber (XL-1000 UV cross-linker, Spectronics corporation, Westbury, NY, USA) with an accumulated dose of 20 J/cm^2^. The UVA light source emits radiation at a range of 320~380 nm with main output at 365 nm. The surface of the mixture was kept at a distance of 3 cm from the filter surface where the light intensity was 2 mW/cm^2^ s (or 20 W/m^2^ s), as measured using a Vilber Lourmat radiometer (Biotronic UV, Vilber Lourmat, Marne La VallCe, France). After irradiation, the cells were further washed once with PBS, and supplemented with new DMEM, then harvested after 24 h for western blot assay [8]. Sham-irradiated cells were treated in the same manner except that they were not irradiated.

### 3.3. Measurement of Cell Viability

The cytotoxic effect of Lycogen™ on cell viability was estimated by MTT assay, as described previously [35]. Cells were cultured in 24-well plates at 1 × 10^4^ cells/well in DMEM for 24 h, and each well was washed and incubated with 1 mL of DMEM containing various concentration of Lycogen™ at 37 ºC for another 24 to 72 h. After irradiation, the cells were further washed once with PBS, and supplemented with new DMEM, than harvested after 24 h for cell viability. Each well was then incubated with MTT for 1 h, after which the liquid was removed, and DMSO was added to dissolve the solid residue. The optical density at 570 nm of each well was then determined by using a microplate reader (FLUOstar OPTIMA, BMG Labtech GmbH, Germany).

### 3.4. Measurement of Lipid Peroxidation

Lipid peroxidation was measured as thiobarbituric acid-reactive substances (TBARS) released into the DMEM medium from Hs68 cells following centrifugation at 1000*g* for 10 min. TBARS were measured by mixing equal volumes of the supernatant with 0.7% TBA reagent and 2.5% TCA. BHT (0.5 mM) was added to prevent sporadic lipid peroxidation during heating at 100 ºC for 10 min. TBARS were extracted with an equal volume (3 mL) of butanol. After a brief centrifugation, the fluorescence of the butanol layer was measured at 515 nm excitation and 555 nm emissions [36]. TBARS were expressed as nmol malondialdehyde (MDA) equivalent/mg protein using 1,1,3,3-tetramethoxypropane as MDA standard.

### 3.5. Western Blot Assay

The treated cells were harvested and lysed with 20% SDS containing 1 mM phenylmethylsulfonyl fluoride. The lysate was sonicated for 1 min on ice followed by centrifugation at 12,000*g* for 30 min at 4 ºC. Mitochondrial and cytosolic fractions were isolated by using the ProteoExtract^®^ Cytosol/Mitochondria Fractionation Kit (Merck Millipore, Billerica, MA, USA). Then a sample of protein from the supernatant was resolved by SDS-PAGE and transferred onto a nitrocellulose membrane. After blocking with TBS buffer (20 mM Tris-HCl, 150 mM NaCl, pH 7.4) containing 5% nonfat milk, the membrane was incubated with antibodies against type I procollagen, MMP-1 (Santa Cruz Biotechnology, Santa Cruz, CA, USA), followed by horseradish peroxidase-conjugated secondary antibodies and then was visualized with an ECL chemiluminescence detection kit (PerkinElmer Life Sciences, Waltham, MA, USA). The relative density of the immunoreactive bands was quantified by using a luminescent image analyzer (LSA-100, Fujifilm, Japan).

### 3.6. Measurement of NFκB

To determine the expression of NFκB, Hs68 cells were treated with Lycogen™, for 24 h, followed by washing with PBS before incubation with 20 J/cm^2^ UVA. To detect NFκB, the medium was collected. Levels of NFκB in the medium were determined by an enzyme-linked immunosorbent assay (ELISA, R&D, Minneapolis, MN, USA).

### 3.7. Data Analysis

Results were expressed as mean ± SD. Statistical analyses were performed by using one-way analysis of variance followed by Duncan’s multiple range tests. Results were considered statistically significant at *p* < 0.05.

## 4. Conclusions

Skin aging is a complex process influenced by many factors, and UV-light exposure, especially UVA, is the most common factor. Although lycopene has been known as a crucial photoprotectant in skin health, several limitations in the extraction methodology of lycopene has hindered the development and clinical utility of lycopene. It is therefore urgent to identify novel bioactive compounds that exhibit functions similar to that of lycopene. In this study, our findings demonstrated that chronic exposure to Lycogen™, a bacteria-derived compound structurally similar to lycopene, was able to prevent the downregulation of procollagen I, intracellular accumulation of MDA, induction of MMP-1 and elevated NFκB levels, which were elicited by UVA exposure. These data suggest that Lycogen™ has high potential as a promising ingredient in cosmetic and pharmaceutical applications.
